# Genomic heterogeneity within B/T mixed phenotype acute leukemia in a context of an immunophenotype

**DOI:** 10.1016/j.lrr.2023.100410

**Published:** 2023-12-31

**Authors:** Ruifang Zheng, Franklin Fuda, Jeffrey R. Gagan, Olga K. Weinberg, Prasad Koduru, Miguel Cantu, Kathleen Ludwig, Jamie M. Truscott, Robert Collins, Stephen Chung, Yazan F. Madanat, Weina Chen

**Affiliations:** a Departments of Pathology, University of Texas Southwestern Medical Center, Dallas, TX 75390, USA; b Departments of Pediatrics (Hematology and Oncology), University of Texas Southwestern Medical Center, Dallas, TX 75390, USA; c Internal Medicine, University of Texas Southwestern Medical Center, Dallas, TX 75390, USA

**Keywords:** Mixed phenotype acute leukemia, B/T MPAL, Immunophenotype, Genotype, JAK/STAT pathway, RAS pathway, NOTCH1, PHF6, AML, T-ALL, B-ALL

## Abstract

B/T mixed phenotype acute leukemia (MPAL) is a rare aggressive leukemia. Three cases of B/T MPAL were identified with comprehensive immunophenotypic, cytogenetic, and molecular studies. T-lineage predominant B/T MPAL shares a genetic signature with T-ALL whereas B/T lineage co-dominant B/T MPAL lacks such a T-ALL signature*.* All three patients were treated with lineage-matched-ALL therapy and alive at the last follow-up. Our study is the first to demonstrate molecular heterogeneity within B/T MPAL in a context of an immunophenotype of T-lineage versus B-lineage predominance. The implication of such a phenotype-genotype association on diagnostic classification is briefly discussed.

## Highlights

• Molecular heterogeneity within B/T mixed phenotype acute leukemia (MPAL) in the context of an immunophenotype.
• T-lineage predominant B/T MPAL sharing the genetic features of T-ALL.
• Patients generally respond well to lineage matched treatment.
• Future studies to explore phenotype-genotype associations and its implication on diagnosis and treatment choice are needed.

## 1 Introduction

Mixed phenotype acute leukemia (MPAL) is a heterogenous group of aggressive acute leukemia (AL) where the blast population(s) express more than one type of lineage-defining marker and the leukemia cannot be classified into other clinically or genetically defined categories such as acute myeloid leukemia (AML) with myelodysplasia-related changes (AML-MR) by the WHO classification, including the recently published 5th WHO classification and the International Consensus Classification (ICC) [1], [2], [3], [4], [5]. AML-MR includes cases with myelodysplasia-related cytogenetic abnormalities (including a complex karyotype) and gene mutations. Diagnosis and clinical management of MPAL are often challenging due to rarity of the disease (accounting for ∼3% of AL), complexity of the lineage marker expression with associated evolving diagnostic criteria, and genomic diversity.

Of MPAL subtypes, B/T MPAL with the blast population co-expressing both B- and T-cell lineage defining markers is exceedingly rare and accounts for ∼3% of MPAL [6], [7], [8], [9]. Only ∼30 cases have been reported in the literature with comprehensive clinicopathological and molecular characterization [7], [8], [9], [10], [11]. B/T MPAL typically occurs in children, adolescents, and young adults with a male predominance. Involvement of bone marrow (BM), peripheral blood (PB), and extramedullary organs (mostly lymph nodes) are common. Most reported cases have cytogenetic abnormalities (∼70–80%) with a subset (∼20–40%) harboring complex karyotype. A complex karyotype in this setting poses a diagnostic dilemma as to whether the leukemia meets criteria for MPAL or AML-MR. Recurrent genetic aberrations include variants in *PHF6, NOTCH1,*  in genes in the JAK-STAT (*JAK3, IL7R*) and the RAS (*NRAS, PTPN11)* pathways, and gene fusions of *SET::NUP214, KTM2A::ELL*, and *CRBN::GABRB2*.

While the aforementioned studies suggest that genetic aberrations of B/T MPAL are similar to those of T-lymphoblastic leukemia/lymphoma (T-ALL), given its rarity with only a few reported studies, we sought to investigate the clinicopathologic and genomic characteristics of 3 cases of B/T MPAL at our institution. We were particularly interested in exploring whether the genomic landscape is associated with an immunophenotype of T-lineage or B-lineage predominance given the distinctly different pathogenesis between T-ALL and B-lymphoblastic leukemia/lymphoma (B-ALL). Evaluation of these genotype-phenotype (lineage predominance) associations in B/T MPAL has not yet been systematically performed. Our study is the first to apply the 5th WHO and ICC classifications in diagnosis and to demonstrate molecular heterogeneity within B/T MPAL in a context of T-lineage or B-lineage predominance. Specifically, T-lineage predominant B/T MAPL shares a genetic signature of T-ALL whereas B/T lineage co-dominant B/T MPAL lacks such a T-ALL signature. Furthermore, we report a previously underreported *SFPQ::ZFP36L2* fusion in B/T MPAL and a novel *NUP98::MLLT1* fusion*,* thus providing further insight into MPAL pathogenesis. Lastly, we discuss the challenges in applying the MPAL diagnostic criteria as to whether the cases with immunophenotypically defined MPAL and carrying myelodysplasia-related genetic changes should be diagnosed as MPAL or AML-MR.

## 2 Material and methods

### 2.1 Clinical and laboratory data

We identified 3 cases of *de novo* newly diagnosed B/T MPAL with comprehensive flow cytometric immunophenotypes, cytogenetic studies, and next generation sequencing (NGS) between 2019 and 2022. The clinical and laboratory data of the patients (Table 1) were retrieved from the electronic medical record system in accordance with an IRB-approved protocol.

**Table 1:** Clinicopathological features of B/T MPAL.

	**Case 1**	**Case 2**	**Case 3**
**Age (year)**	7	41	19
**Gender**	M	M	M
**Clinical presentation**	Rash, night sweats, firm, non-tender lymphadenopathy	Prominent lymphadenopathy	Syncope due to pancytopenia
**WBC (K/μL)**	100.8	6.98	1.4
**Hb (g/L)**	8.1	14.5	2.7
**Plt (K/μL)**	68	111	251
**PB blast%**	76 %	0 %	0.50 %
**BM Blast%**	89 %	6 %	52 %
**BM involvement**	Y (extensive)	Y (mild)	Y (extensive)
**Extra medullary involvement (LA)**	Y	Y (predominant)	Y
**B/T lineage predominant**	T	T	B/T
**Chemotherapy**	T-ALL therapy (AALL1231, AALL0434 with nelarabine)	T-ALL therapy (AALL0434)	B-ALL therapy (AALL1732)
**Response at end of induction in BM**	Negative for MRD (<0.01 %)	Negative for MRD (<0.01 %)	Negative for MRD (<0.01 %)
**in LN**		CR	
**BM transplant**	N	Y	N
**Follow-up (f/u) (months)**	36	12	27
**Alive (A)**	A/CR1	A/CR1	A/CR1

MRD: minimal residual disease in BM detected by flow cytometry at day 29; N, no; Y, yes; M, male; WBC, white cell count; Hb, hemoglobin; Plt, platelet, PB, peripheral blood; BM, bone marrow; LA, lymphadenopathy; LN, lymph node; CR1, first complete remission.

### 2.2 Morphology and flow cytometry

Wright-Giemsa-stained peripheral blood and bone marrow aspirate smears and hematoxylin/eosin-stained trephine biopsy/aspirate clot sections and lymph nodes were reviewed. Flow cytometric analyses were performed on bone marrow aspirate, peripheral blood, or lymphoid tissue on the 10-color BD FACS Canto instruments (Becton Dickinson) and analyzed using cluster analysis with Cytopaint Classic software (Leukobyte) as described preciously [12]. A panel of lymphoid and myelomonocytic markers (Table 2) was used for comprehensive immunophenotypic analysis. A total of 100,000 events/tube was collected.

**Table 2:** Immunophenotype of B/T MPAL by flow cytometric analysis.

	**Case 1**		**Case 2**	**Case 3**
	**(bilineal/**	**biphenotypic)**	**(biphenotypic)**	**(biphenotypic)**
**Lineage of predominant blasts**	**T-lineage**		**T-lineage**	**B/T-lineage**
**Lymphoblast%**	B/T blasts: 59 %	T-lymphoblasts: 19 %	B/T blasts: 91 %	B/T blasts: 66 %
**Heterogenous antigen expression**	yes	NA	yes	yes
**B-lineage markers**				
CD19	subset (4 %) strong +	-	subset strong +	subset strong +
CD79a	subset strong +	partial dim +	subset strong +	partial +
CD22	partial +, 60 %	predominantly -	subset strong +	subset strong +
CD10	few dim +	-	-	-
CD20	subset +, 4 %	-	-	partial dim +
sIg	-	-	-	-
**T lineage markers**				
cCD3	strong +	strong +	subset strong +	subset strong +
sCD3	dim + variable	+	-	-
CD1a	-	partial +	–	–
CD2	-	partial +	+	-
CD4	-	-	-	-
CD5	-	dim +	partial +	partial +
CD7	+	+	+	+
CD8	-	-	-	-
**ETP-ALL-like IP**	Yes	no	yes	yes
**Myeloid/monocytic markers**				
MPO	-	-	-	-
CD13	+	-	-	–
CD14	-	-	-	-
CD15	-	-	-	-
CD33	-	-	partial +	-
CD117	–	-	-	partial +
CD11b	-	+	partial +	partial +
CD64	-	-	-	-
**Immature markers**				
CD34	1 %+	-	small subset +	-
TdT	+	-	+	+
**Other markers**				
CD25	-	-	subset +	subset dim +
CD38	+	+	+	+
CD45	dim +	+	+	-
CD56	-	-	+	-
CD123	–	–	dim +	dim +
HLA-DR	-	-	partial +	+

ETP-ALL-like IP: early T-cell precursor ALL (ETP-ALL)-like immunophenotype; c, cytoplasmic; s, surface.

All cases demonstrated a pattern of heterogenous antigen expression (e.g., coordinated strong co-expression of B-cell markers (CD19 and CD22) on a subset of blasts in case 2.

B/T MPAL was diagnosed per the 5th WHO/ICC classifications for hematolymphoid tumors [3]. Specifically, strong antigen expressions of B-lineage defining markers (CD19, CD22, and CD10) and T-lineage defining markers (surface CD3 and intracellular CD3) were defined as the brightest antigen expression in a population of blasts exceeding an intensity level of 50 % of normal mature B cells and T cells, respectively. In this study, the brightest antigen expression in all cases reached the intensity of their normal counterparts. Additionally, all cases exhibited a pattern of heterogenous antigen expression.

### 2.3 Chromosomal and FISH analyses

Karyotypic analysis was performed on Giemsa-banded metaphase cells from bone marrow aspirates using standard techniques. Fluorescence in situ hybridization (FISH) was performed on either bone marrow aspirates or lymphoid tissue using standard techniques. FISH panel details are presented in the footnote of Table 3.

**Table 3:** Cytogenetic and molecular findings of B/T MPAL.

FISH panel covers high risk lymphoblastic leukemia, myelodysplastic syndrome, and large B-cell lymphoma. Key.

genes/fusions include *CRLF2, ABL1, BCL6,* 4cen/10cen, *PDGFRA::FIP1L1, PDGFRB, FGFR1*, 8cen/20q12,.

*MYC* BA, *MYC::IGH, BCR::ABL1, KMT2A*(MLL), *ETV6::RUNX1*, and *IGH::BCL2*.

### 2.4 Next generation sequencing

DNA and RNA were isolated from either fresh bone marrow (cases 1 and 3) or lymph node (case 2) with paired germline specimens from saliva. A custom institutional next generation sequencing (NGS) panel with 138 cancer-related genes in DNA and 1526 genes in RNA was performed on Illumina NextSeq 550 instruments. The variant allele frequency limit of detection was 5 % for single nucleotide variants and 10 % for indels. RNA sequencing required 10 split or discordant reads for gene fusions. Full details of the genes tested, exon coverage, and the bioinformatics pipeline are available at http://www.utsouthwestern.edu/sites/genomics-molecular-pathology/.

## 3 Case presentation

### 3.1 Case 1

**Fig. 1:**
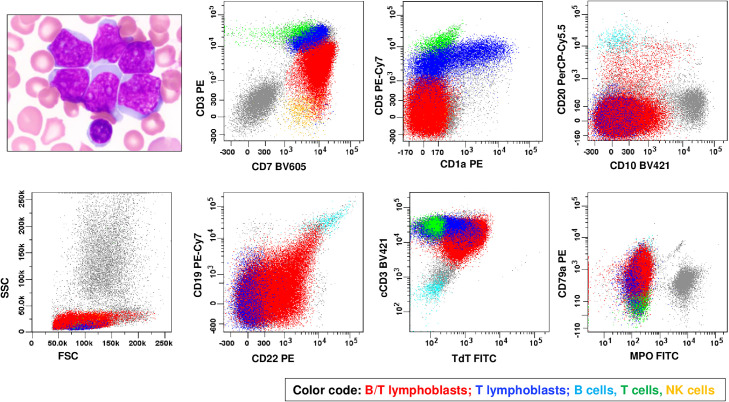
Morphology and immunophenotype of a case of T-lineage predominant B/T MPAL (bilineal/biphenotypic) by flow cytometry on BM aspirate (case 1). Medium-sized to large lymphoblasts with occasionally irregular nuclei, moderately dispersed chromatin, inconspicuous nucleoli, and scant cytoplasm without granules (Wright-Giemsa stain, ×100 objective). By flow cytometry, B/T lymphoblasts (in red) express surface (dim) and cytoplasmic CD3, CD7, CD10(partial), strong CD19/CD20/CD22 [(a small subset, 4 %, approaching to the intensity of normal B cells (in cyan)], CD79a, and TdT, but lack CD1a, CD5, or MPO. T lymphoblasts express surface (dim) and cytoplasmic CD3, CD1a (partial), CD5 (dim), CD7, CD79a (partial), and TdT but lack expression of CD10, CD19, CD20, CD22 or MPO.

A 7-year-old male presented with rash, neck and groin swelling, and night sweats. Physical examination revealed firm, non-tender lymphadenopathy in multiple locations. Laboratory tests revealed significant leukocytosis, anemia, and thrombocytopenia (Table 1). The peripheral blood (PB) and bone marrow (BM) aspirate smears revealed abundant lymphoblasts, medium-sized to large blasts with variably irregular nuclei, inconspicuous to small nucleoli, and scant cytoplasm (Fig. 1). The cerebrospinal fluid (CSF) was also positive for leukemia infiltrate, indicating central nervous system (CNS) involvement. Flow cytometry analysis on a BM aspirate revealed two distinct blast populations. The first population (59%) displayed a B/T lymphoblast phenotype, positive for surface (s) and cytoplasmic (c) CD3, CD19 (small subset strong), CD22, and terminal deoxynucleotidyl transferase (TdT), but negative for myeloperoxidase (MPO) and monocytic markers. The second population (19%) exhibited a T-lymphoblast phenotype, positive for CD1a (partial), sCD3, cCD3, and TdT but negative for CD19 or CD22. This immunophenotype fulfilled the diagnostic criteria for B/T MPAL (bilineal/biphenotypic) with a T-lineage predominance and non-ETP-ALL-like immunophenotype (Table 2). Cytogenetic and NGS studies detected an *SFPQ::ZFP36L2* fusion that corresponded to a t(1;2)(p34;p21) and multiple gene mutations in *IL7R, ARID1A, PHF6, NOTCH1,* and *RUNX1* (Table 3).

The patient was treated with a T-ALL-based protocol and achieved complete remission at the end of induction without minimal residual disease (MRD < 0.01 % by flow cytometry on a BM sample) and was in first complete remission at the last follow-up (36 months after diagnosis) (Table 1).

### 3.2 Case 2

**Fig. 2:**
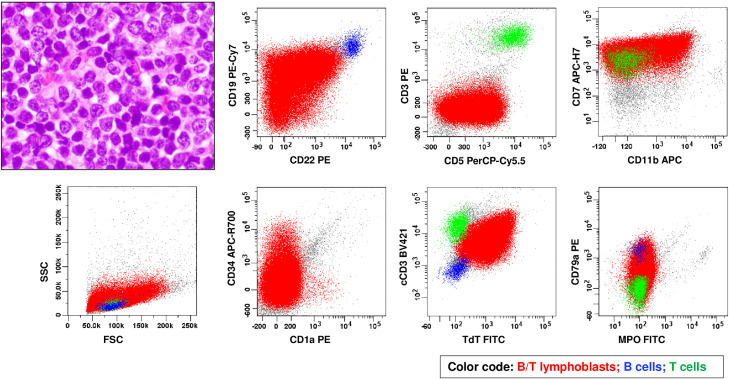
Morphology and immunophenotype of a case of T-lineage predominant B/T MPAL (biphenotypic) by flow cytometry on lymph node (case 2). Diffuse infiltrate of medium-sized to large lymphoblasts with inconspicuous nucleoli and scant cytoplasm [(hematoxylin and eosin (HE) stain, ×40 objective)]. By flow cytometry, B/T lymphoblasts (in red) express cytoplasmic CD3, CD34 (small subset), CD5 (partial), CD7, CD11b (partial), strong CD19/CD22/CD79a (subset with the intensity approaching to the normal B cells in blue), and TdT but largely lack expression of CD1a, surface CD3, or MPO.

A 41-year male presented with an enlarged lymph node in the neck. Imaging studies revealed lymphadenopathy above and below the diaphragm (stage IV). Lymph node biopsy showed a lymphoblast infiltrate (Fig. 2). Flow cytometric analysis on lymph node revealed a T/B lymphoblast population with a T-lineage predominance, positive for cCD3, CD19/CD22 (subset strong), but negative for CD1a, sCD3, MPO or monocytic markers (Table 2). This immunophenotype fulfilled the diagnostic criteria for B/T MPAL (biphenotypic) with a T-lineage predominance and ETP-ALL-like immunophenotype. BM marrow was involved by B/T MPAL with a low percentage of lymphoblasts (5.3%). Cytogenetic study revealed a normal male karyotype. NGS identified multiple gene mutations in *JAK3, STAT5B, PHF6, NRAS*, and *RUNX1* (Table 3).

The patient was initiated on T-ALL therapy following the CALGB 10403 protocol and achieved partial remission with lymph node involvement at the end of induction (despite MRD negative in BM) and achieved complete remission with no lymph node involvement at the end of the second course of chemotherapy. He underwent allogeneic hematopoietic stem cell transplantation (allo-HSCT) and remained in complete remission at the last follow-up (12 months after diagnosis) (Table 1).

### 3.3 Case 3

**Fig. 3:**
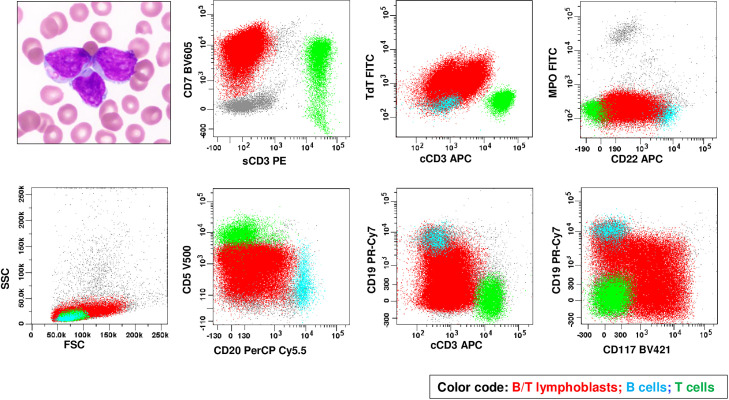
Morphology and immunophenotype of a case of B/T-lineage co-dominant B/T MPAL (biphenotypic) by flow cytometry on BM aspirate (case 3). Medium-sized to large lymphoblasts with occasionally irregular nuclei, moderately dispersed chromatin, inconspicuous nucleoli, and scant cytoplasm with occasional vacuoles and granules (Wright-Giemsa stain, ×100 objective). By flow cytometry, B/T lymphoblasts (in red) express cytoplasmic CD3 (subset strong), CD5 (partial), CD7, strong CD19/CD20/CD22 (subset with the intensity approaching to the normal B cells in cyan), CD117 (partial), and TdT but lack expression of surface CD3, or MPO.

A 20-year-old male presented with syncope and was found to have pancytopenia. Imaging studies revealed lymphadenopathy in the cervical and upper abdominal regions. Laboratory tests were notable for pancytopenia, rare blasts (0.5%) in PB and a significant lymphoblast infiltrate (52 %) in BM aspirate (Fig. 3) (Table 1). Flow cytometric analysis on a BM aspirate demonstrated a lymphoblast population with B-lineage and T-lineage co-dominance, positive for cCD3 and CD19/CD22 (subset strong), but negative for CD1a, sCD3, MPO and monocytic markers. This immunophenotype met the diagnostic criteria for B/T MPAL (biphenotypic) with a B-/T-lineage co-dominance and ETP-ALL-like immunophenotype (Table 2). Cytogenetic and FISH analyses revealed a complex karyotype: 46,XY,del(6)(q13q23),t(7;9)(p13;q34),del(12)(p12p13) and *NUP98* (11p15.4) rearrangement. NGS study revealed a *NUP98::MLLT1* fusion that would correspond to a t(11;19)(p15;p13) and gene mutations in *NF1* and *BCOR* (Table 3).

The patient was treated with B-ALL-based protocol and achieved complete remission at the end of induction without minimal residual disease (MRD < 0.01 % by flow cytometry on BM sample) and was in first complete remission at the last follow-up (27 month after diagnosis).

## 4 Discussion

This is the first study demonstrating molecular heterogeneity within B/T MPAL in a context of an immunophenotype of T-lineage or B-lineage predominance using the 5th WHO and ICC diagnostic criteria. Specifically, T-lineage predominant B/T MPAL shares a genetic signature with T-ALL, whereas B/T lineage co-dominant B/T MPAL lacks such a T-ALL signature. Importantly, all three patients responded well to lineage-matched ALL-based therapy.

Our study confirms previously characterized clinicopathological and molecular features of B/T MPAL, such as commonly occurring in adolescents and young adults (a median age of 19 years in our study) with a male predominance (3/3 males in our study), frequent involvement of PB/BM and extramedullary organs/lymph nodes (3/3 in our study), cytogenetic abnormalities (70–80%, 2/3 in our study) with a subset harboring complex karyotype (20–40%, 1/3 in our study), and recurrent genetic aberrations commonly present in T-ALL (2/3 in ours) [[7], [8], [9], [10], [11],^13^].

Such T-ALL-like genetic alterations include mutations in *PHF6, NOTCH1,* and in genes involving the JAK-STAT (*JAK3* and *IL7R*) and the RAS (*NRAS, PTPN11*, and *NF1)* pathways*,* as well as fusion of *SET::NUP214.* Notably, this genetic profile was present in the two cases of B/T MPAL with a T-lineage predominance in our study. Intriguingly, our study also highlights a previously underreported finding of B/T-lineage co-dominant MPAL harboring genetic aberrations distinct from those of T-ALL; this case (patient 3) harbored mutations in *NF1* and *BCOR1* along with *NUP98::MLLT1*. Our results suggest molecular heterogeneity within B/T MPAL in a context of an immunophenotype of T-lineage versus B-lineage predominance, which is not entirely unexpected given the distinctly different pathogenesis of T-ALL versus B-ALL [3,14]. Indeed, one reported case of B/T MPAL (1/9) without specifics on the T-lineage or B-lineage predominance had *IKZF1* mutation [10], a common alteration in Philadelphia chromosome-like (Ph-like) B-ALL [15].

There are previously underreported and novel genetic alterations in our cohort that merit further discussion. In the two cases of T-lineage predominant B/T MPAL, we identified alterations in *STAT5B* and *SFPQ::ZFP36L2* which have been previously reported in a small subset of T-ALL (*SFPQ::ZFP36L2* being extremely rare) [11,16,17] but have not yet been reported in B/T MPAL. Concurrent mutations in *RUNX1* and *PHF6* along with *NOTCH1* mutation indicate an alteration of early hematopoietic differentiation to promote T-cell differentiation at the expense of B-cell differentiation. As mentioned, our case of B/T-lineage co-dominant MPAL harbored *NF1* and *BCOR1* mutations and *NUP98::MLLT1. NF1* is commonly mutated in B-ALL and reported in one case of B/T MPAL [9,18] while *BCOR* is typically mutated in myeloid neoplasms [19] and has not yet been reported in B/T MPAL. Interestingly, *NUP98::MLLT1* is a novel fusion that has not yet been characterized. *NUP98* rearrangement (r) is present in AML [20] and rarely in B/M MPAL with *NUP98::NSD1* [7,21], and *MLLT1* is a recurrent partner of *KMT2A*-r in acute leukemias*.* Additionally, *NUP98* fusions are associated with *HOX* gene/protein upregulation, especially *HOXA* [22,23]. Other genetic lesions resulting in *HOXA* dysregulation include *KMT2A*-r and *SET::NUP214* [14,24]. These associations suggest that *NUP98::MLLT1* in our case of B/T MPAL is likely an important pathogenetic alteration with mechanism(s) similar to those conferred by *SET::NUP214* and *KTM2A*-r (including *KTM2A::ELL*), as previously reported in 3 cases of B/T MPAL [7,9,10]. Collectively, our study not only confirms reported genetic alterations in B/T MPAL but also expands the genomic landscape through identification of additional molecular abnormalities, providing further insights into B/T MPAL pathogenesis.

Importantly, while our finding of a phenotype-genotype association within B/T MPAL in a small cohort requires further investigation of genomic aberrations in the context of lineage predominance, such association may have implications on diagnosis and treatment. Current investigative studies on the genomic landscape of MPALs mainly focus on genetically defined entities, such as *KMT2A-r, BCR::ABL1*, the recently added *ZNF384-r* and *BCL11B*-activated MPAL, and generic MPAL-NOS (not otherwise specified, by immunophenotype) without providing immunophenotypic specifics on which lineage predominates [[7], [8], [9], [10], [11],^21^,^25^,^26^]. Likewise, MPAL diagnosis may not include such immunophenotypic detail. We strongly advocate for including immunophenotypic-lineage predominant information in clinical MPAL diagnosis and in research studies for two main reasons. First, molecularly defined MPAL may have heterogeneous genetic features, e.g., *BCR::ABL1* MPAL encompassing two genetic subgroups (i.e., a G5 subtype with an AML-like gene expression profile and a G8 subtype with common lymphoid progenitor disease-like signatures) [9]. Second, lineage assignment is a fundamental attribute in tumor classification [3] and many tumor regimens are tailored to lineage assignment while genomic/molecular attributes are important for tumor biology and therapeutic targets. It has been shown that MPAL patients who received lineage-matched therapy have a better clinical remission rate compared to those who did not [21]. The importance of lineage matched therapy is supported by the fact that all three patients in our cohort with B/T MPAL achieved complete remission after receiving lineage-matched ALL-based therapy. Following current treatment guidelines [25], one adult patient in first complete remission (CR1) underwent allo-HSCT while allo-HSCT for the two pediatric patients in CR1 was not indicated. These data advocate that clinical diagnosis and investigative studies of MPAL should include phenotypic description along with lineage predominance as well as genetic information.

Lastly, myelodysplasia-related cytogenetic and gene mutations in cases of immunophenotypically defined B/T MPAL raise diagnostic challenges. Complex karyotype, myelodysplasia-associated chromosomal abnormalities (-5q, +8), and myelodysplasia-associated gene mutations (as defined by 5th WHO and ICC, such as mutations in *ASXL1, BCOR, RUNX1, SF3B1, U2AF1*, and *EZH2*) have been reported in ∼30%, ∼10–20%, ∼30% of B/T MPAL cases, respectively [7], [8], [9], [10], [11]. The 3 cases of B/T MPAL in our cohort harbored such genetic/molecular profiles (complex karyotype, mutations in *BCOR* and *RUNX1*, one in each case). These scenarios create diagnostic controversy as to whether these cases with immunophenotypically defined B/T MPAL (but without evidence of strong myeloid differentiation) and carrying myelodysplasia-related genetic changes should be diagnosed as MPAL or AML-MR. This diagnostic challenge was also discussed in the two recent reviews [27,28]. Under the current diagnostic guidelines by the 5th WHO and the ICC [3], [4], [5], such genetic profiles necessitate a diagnosis of AML-MR rather than B/T MPAL. Notably, emerging evidence would argue for a diagnosis of B/T MPAL for multiple reasons. First, similar to our cohort, no lineage defining myeloid markers were expressed in any of these reported cases of B/T MPAL harboring myelodysplasia related genetic abnormalities. Second, such B/T MPAL patients generally respond well to ALL-based regimens. Third, myelodysplasia-associated gene mutations are not specific for AML-MR and present in ∼30% of *de novo* AML cases without myelodysplasia-related changes in a recent study [29]. Furthermore, these mutations are not even specific for AML, e.g., *EZH2, ASXL1*, and *RUNX1* mutations are present in ∼5% of T-ALL and in ∼2% of B-ALL [30,31]. Fourth, nearly all recent genomic/clinical studies that have defined MPAL in the last 5 years since publication of the 2017 WHO classification include those cases with so-called myelodysplasia defining cytogenetic/molecular features [[7], [8], [9], [10], [11],^21^,^25^,^26^]; this apparent reluctance to fully apply the WHO criteria is a testament to this unsettled debate. Future well-designed studies to include comprehensive phenotypic features with assessment of lineage predominance as well as genetic features are imperative to reconcile diagnostic controversy and refine MPAL diagnostic criteria.

In summary, our study demonstrates molecular heterogeneity within B/T MPAL in a context of an immunophenotype of T-lineage or B-lineage predominance in a cohort of 3 cases. Future studies are needed to evaluate phenotypic and genotypic associations in MPAL including assessing lineage-matched treatment in the context of genetics. A better understanding of MPAL biology is needed to clarify diagnostic ambiguity and to refine diagnostic criteria centered on myelodysplasia-associated cytogenetics and gene mutations.

## Funding

None.

## Ethics statement

This study was approved by the institutional review board. Ethics approval and/or informed consent were not required for this study.

## Informed consent

This study was approved by Institutional Review Board. Informed consent was not required per the study protocol.

## CRediT authorship contribution statement

**Ruifang Zheng:** Conceptualization, Writing – original draft, Data curation, Formal analysis. **Franklin Fuda:** Data curation, Writing – review & editing. **Jeffrey R. Gagan:** Data curation, Investigation, Methodology. **Olga K. Weinberg:** Data curation. **Prasad Koduru:** Data curation, Methodology. **Miguel Cantu:** Data curation. **Kathleen Ludwig:** Data curation. **Jamie M. Truscott:** Data curation. **Robert Collins:** Data curation. **Stephen Chung:** Data curation. **Yazan F. Madanat:** Data curation. **Weina Chen:** Conceptualization, Data curation, Formal analysis, Investigation, Writing – original draft, Writing – review & editing.

## Declaration of competing interest

Y.F.M. has received honoraria/consulting fees from BluePrint Medicines, GERON, OncLive and MD Education. Y.F.M. participated in advisory boards and received honoraria from Sierra Oncology, Stemline Therapeutics, Blueprint Medicines, Morphosys, Taiho Oncology and Novartis. Y.F.M. received travel reimbursement from Blueprint Medicines and Morphosys. None of these relationships were related to this work. The remaining authors declared no conflict of interest.
